# Case Report: Primary splenic angiosarcoma: a case report and literature review

**DOI:** 10.3389/fonc.2026.1927091

**Published:** 2026-10-19

**Authors:** Jinqin Zha, Hang Zhou, Hao Yin, Zan Zhou, Xinkai Xia, Hailong Zhao, Rongchun Xing

**Affiliations:** 1 The First College of Clinical Medical Science, China Three Gorges University, Yichang, Hubei, China; 2 Department of General Practice, Yichang Central People’s Hospital, Yichang, Hubei, China; 3 Department of Hepatobiliary Surgery, Yichang Central People’s Hospital, Yichang, Hubei, China; 4 Department of Pathology, Yichang Central People’s Hospital, Yichang, Hubei, China; 5 Department of Endocrinology, Yichang Central People’s Hospital, Yichang, Hubei, China; 6 Department of General Surgery, Jinggangshan University Affiliated Hospital, Ji’an, Jiangxi, China

**Keywords:** anlotinib, case report, sintilimab, spleen, splenectomy, splenic angiosarcoma, splenic rupture, vascular tumor

## Abstract

Primary splenic angiosarcoma (PSA) is a rare, highly aggressive malignant vascular tumor with nonspecific clinical and imaging findings. We report a 42-year-old man who presented with recurrent left upper abdominal pain after splenic artery embolization for multiple hypervascular splenic lesions with local rupture and hemorrhage. Because symptoms recurred, imaging showed a complex splenic lesion, and the diagnosis remained uncertain, open splenectomy and lysis of intestinal adhesions were performed on March 12, 2024. Intraoperatively, approximately 4000 mL of bloody ascites and active splenic bleeding were found. Pathology showed destruction of the splenic architecture by anastomosing abnormal vascular channels, intraluminal papillary structures, and focal solid areas, with marked atypia, extensive necrosis, a Ki-67 labeling index of approximately 40%, mutant-type p53 expression, and positivity for CD31, FLI-1, and ERG, supporting splenic angiosarcoma. After surgery, the patient was clinically managed as having advanced disease with hepatic and bone involvement and received denosumab, first-line EIM chemotherapy, second-line gemcitabine plus albumin-bound paclitaxel combined with anlotinib, followed by the addition of sintilimab, and subsequent maintenance therapy with anlotinib plus sintilimab. The medical record documented progressive disease after two cycles of EIM therapy and repeated stable disease during later treatment. The formal imaging reports also described mixed objective changes, including partial shrinkage of hepatic lesions at several time points. The last treatment and follow-up date supported by the available records was October 10, 2025. This case suggests that PSA should be considered early when complex vascular splenic lesions are accompanied by splenomegaly, recurrent pain, anemia, and bleeding or rupture risk. Splenectomy can provide both diagnostic and therapeutic value, while individualized multimodal treatment and close follow-up remain central for advanced PSA.

## 1 Introduction

PSA arises from splenic vascular endothelium or splenic sinus-related endothelial cells. It is one of the rarest primary malignant tumors of the spleen. Previous world reviews, pathology series, Surveillance, Epidemiology, and End Results (SEER) studies, and recent case reports show that PSA may present with abdominal pain, splenomegaly, anemia, fatigue, weight loss, and spontaneous splenic rupture. The liver, lung, bone, and bone marrow are common sites of involvement. The overall prognosis is poor (1–8).

Littoral cell-related tumors form a vascular tumor spectrum unique to the spleen. Falk et al. first described littoral cell angioma (LCA) and proposed that it arises from littoral cells of the splenic red pulp and shows dual endothelial and histiocytic differentiation (9). Later studies further summarized the immunophenotype and key differential diagnostic features of LCA (10, 11). Littoral cell angiosarcoma (LCAS) and metastasizing littoral cell hemangioendothelioma have also been reported. These reports indicate that tumors within this spectrum may show malignant or low-grade malignant behavior (12–18).

In the present case, the patient first presented with multiple hypervascular splenic lesions and local rupture with hemorrhage. Preoperative CT/CTA favored a vascular lesion, whereas PET/CT once suggested possible lymphoma. Ultimately, pathology of the splenectomy specimen supported splenic angiosarcoma. The patient received multiple lines of systemic therapy after surgery. The medical record showed PD after first-line EIM therapy and repeated assessments of SD during second-line treatment and later targeted therapy combined with immunotherapy. Formal imaging reports, however, described mixed changes at some time points, including lesion shrinkage, enlargement, or increased number. This report describes the case and discusses the key diagnostic and therapeutic issues in light of the literature, with the aim of providing a reference for clinicians who may encounter similar patients.

## 2 Case presentation

### 2.1 General information

A 42-year-old man was admitted on February 20, 2024 because of left upper abdominal pain for half a month after percutaneous splenic artery embolization performed more than 2 months earlier. More than 2 months before this admission, the patient developed abdominal pain without an obvious trigger. He was hospitalized at our institution on December 11, 2023. On December 14, 2023, non-contrast and contrast-enhanced CT of the hepatobiliary system, spleen, and pancreas suggested multiple splenic hemangiomas with local rupture and hemorrhage, dilation of the portal vein and splenic vein, splenomegaly, and decreased splenic density. Multiple hemangioma-like hepatic lesions were also seen. Metastasis could not be excluded, and the lesions were subsequently assessed as metastatic. A right renal cyst and ascites were also present. On the same day, the patient underwent emergency percutaneous splenic arteriography and embolization. After anti-infective therapy and symptomatic supportive treatment, his condition stabilized and he was discharged.

Half a month before this admission, the patient again developed persistent dull pain in the left upper abdomen. The pain worsened after eating greasy food and was accompanied by nausea and vomiting of gastric contents. He had no fever, chills, or melena. He was admitted to the hepatobiliary and pancreatic surgery department from the outpatient clinic with a diagnosis of a splenic mass. On admission, his body temperature was 36.5 °C, pulse was 100 beats/min, respiratory rate was 18 breaths/min, and blood pressure was 138/100 mmHg. He was alert. The sclerae were not icteric. The abdomen was flat and soft. There was tenderness in the left upper abdomen without rebound tenderness. No abdominal mass was palpated. The liver and spleen were not palpable below the costal margin. Murphy’s sign was negative. No edema was present in the lower limbs. The patient had no history of hypertension, diabetes, coronary heart disease, or hepatitis, and he did not smoke or drink alcohol.

### 2.2 Laboratory tests

On February 21, 2024, routine blood testing showed a red blood cell count of 3.98 × 10^12/L, hemoglobin of 116 g/L, and hematocrit of 35.8%. Biochemical and inflammatory markers showed total protein 63.30 g/L, albumin 37.40 g/L, total bile acids 37.6 μmol/L, alkaline phosphatase 132 U/L, gamma-glutamyl transpeptidase 171 U/L, prealbumin 148 mg/L, and C-reactive protein 20.92 mg/L.

### 2.3 Imaging findings and preoperative diagnostic process

Upper abdominal CT on February 19, 2024 showed post-treatment changes of multiple splenic masses with local rupture and hemorrhage, with possible local infarction. Multiple hemangioma-like or low-density hepatic lesions had increased in number and size compared with previous images, and metastasis could not be excluded. Small patchy low-density areas with possible bone destruction were seen in parts of the thoracolumbar vertebrae, some right ribs, and the right iliac bone. Ascites had decreased compared with previous imaging. Left pleural effusion with partial atelectasis of the left lung and a small right pleural effusion were also present. Whole-body bone scintigraphy on February 22, 2024 showed active bone metabolism in the right third anterior rib and left ribs. Multiple low-density bone lesions throughout the body showed no high uptake and were considered tumor-related lesions.

Hepatic arterial imaging and splenic venous imaging (CTA plus CTV) on February 23, 2024 suggested multiple splenic hemangioma-like lesions with local rupture and hemorrhage, post-splenic artery embolization changes, and a high likelihood of local splenic infarction. Splenomegaly and thickening of the portal vein and splenic vein were present. Multiple hemangioma-like low-density hepatic lesions were also present. Metastasis could not be excluded, and the lesions were subsequently assessed as metastatic. A right renal cyst and left pleural effusion with partial atelectasis of the left lung were also noted. On February 23, 2024, the patient underwent ultrasound-guided left pleural puncture and catheter drainage.

Bone marrow cytology on February 27, 2024 showed active marrow proliferation, with a granulocyte-to-erythrocyte ratio of 1.31:1. Granulocytic cells accounted for 44.0%. Erythroid cells accounted for 33.5%, mainly with increased middle and late erythroblasts. Megakaryocytes were visible, and platelets were scattered or distributed in small clusters. No parasites were seen. The white blood cell differential in peripheral blood showed no obvious abnormality. These findings did not support definite infiltration by a hematologic malignancy.

PET/CT on February 27, 2024 showed marked splenomegaly with increased metabolic activity. Osteolytic bone destruction in the right ribs, some vertebral bodies, and pelvis showed partially increased metabolic activity. Focal increased metabolic activity was also seen in the left pleura. The report considered lymphoma to be highly possible and recommended pathologic confirmation. Slight enlargement and mild metabolic activity of a left cervical level IIa lymph node were also noted. Multiple low-density hepatic lesions did not show high metabolic activity. When combined with enhanced CT, these findings favored multiple hemangioma-like hepatic lesions, but metastasis could not be excluded, and the lesions were subsequently assessed as metastatic. Tortuosity and dilation of the portal vein and splenic vein and left pleural effusion were also present. Superficial lymph node ultrasound on March 5, 2024 showed visible bilateral cervical lymph nodes with unclear architecture, and lymphatic disease remained possible.

The initial admission diagnoses were multiple splenic hemangiomas, multiple hemangioma-like hepatic lesions, and multiple gallbladder polyps. During hospitalization, bone abnormalities, increased pleural metabolic activity, and the PET/CT impression of possible lymphoma shifted the diagnostic focus from a splenic vascular mass to a lymphatic tumor. Bone marrow cytology and subsequent examinations did not provide direct evidence of a hematologic malignancy. Given the history of splenic bleeding and embolization, persistent left upper abdominal pain, increasing complexity of the splenic lesions, and the need for pathologic confirmation, the multidisciplinary team decided to proceed with surgery (**Figure 1**).

**Figure 1:**
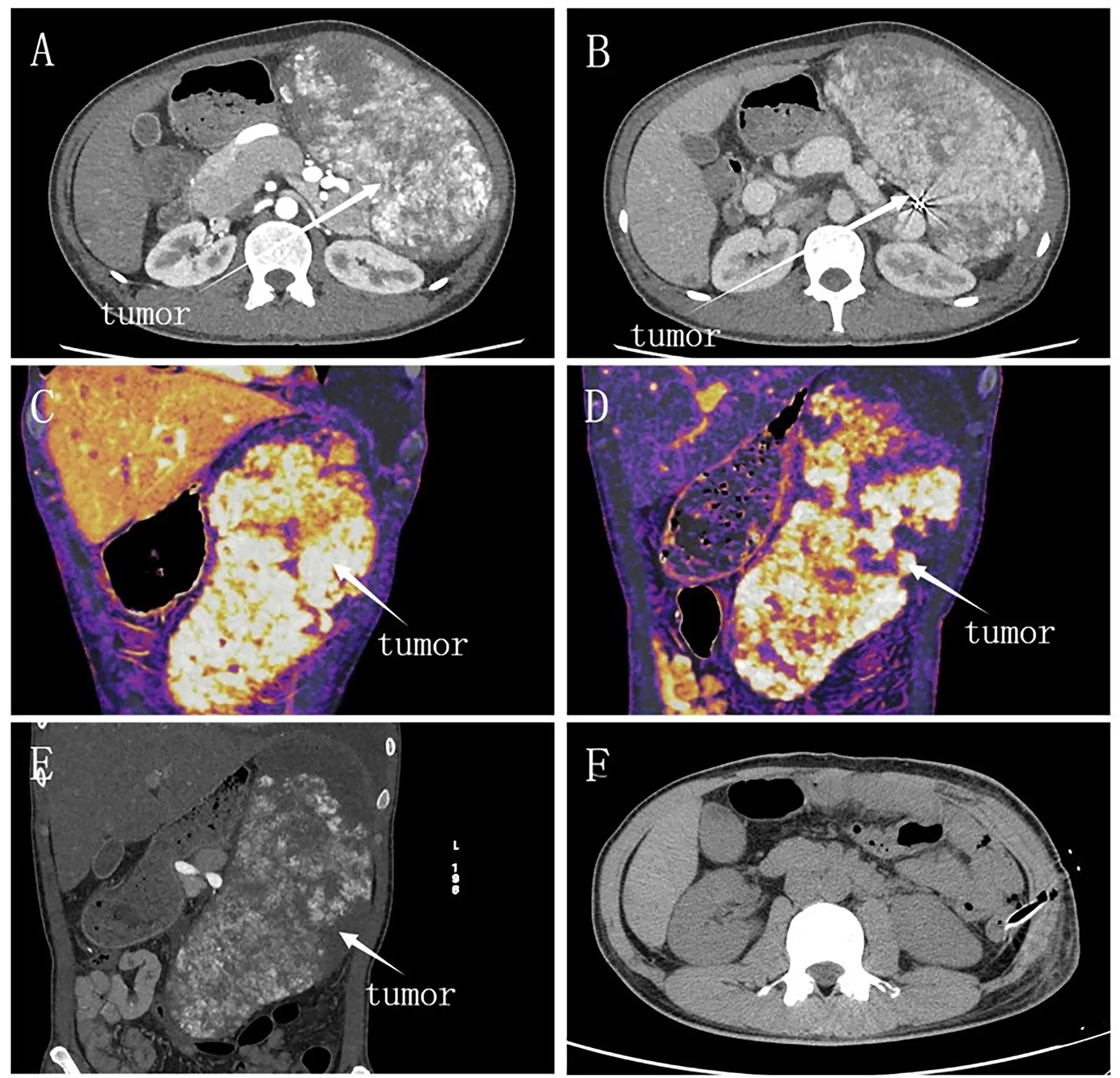
Preoperative and postoperative imaging findings. **(A, B)** Axial contrast-enhanced CT images show marked splenomegaly and a large irregular mass-like lesion in the left upper abdomen. The lesion has markedly heterogeneous internal density and multiple patchy and nodular enhancing areas, suggesting rich blood supply. Internal low-density areas should be considered necrotic, hemorrhagic, or infarct components. The local area of decreased enhancement in panel **(B)** suggests post-embolization change. **(C, D)** Coronal CT fusion images show a large tumor-like lesion in the splenic region with diffuse hypermetabolism and heterogeneous internal uptake, suggesting possible malignancy. **(E)** Coronal contrast-enhanced CT shows a large and infiltrative tumor extent. **(F)** Postoperative CT shows that the spleen has been removed. Drainage tubes, small amounts of gas, and exudation are visible in the left upper abdominal operative area, consistent with postoperative changes after splenectomy.

### 2.4 Surgical treatment

On March 12, 2024, the patient underwent open splenectomy and lysis of intestinal adhesions under general anesthesia. A left subcostal incision of approximately 18 cm was made, and the abdomen was entered layer by layer. Exploration revealed a large volume of fresh bloody intraperitoneal fluid, approximately 4000 mL. The spleen was markedly enlarged, congested, and edematous, with extensive adhesions to surrounding tissues. Adhesions involved the gastrosplenic ligament, splenocolic ligament, and diaphragm. Collateral circulation was seen around the spleen. The splenic artery was markedly thickened, with a diameter of approximately 0.7 cm. The splenic vein was also markedly thickened, with a diameter of approximately 1.2 cm. Active bleeding from the spleen was observed. After the intraperitoneal blood was aspirated, intraoperative autologous blood transfusion was performed. The surgeons gradually mobilized the spleen, ligated and divided the splenic artery, splenic vein, and short gastric vessels, and removed the spleen after dissecting the splenocolic and splenorenal ligaments. The splenic bed and a small area of the pancreatic tail were sutured for hemostasis. During the operation, the patient received 3.5 units of packed red blood cells, 400 mL of plasma, and 10 units of cryoprecipitate. No intraoperative coagulation testing was performed. Preoperative thromboelastography on February 21, 2024 was within the reference ranges (R 5.5 min, K 1.4 min, angle 70.4°, MA 66.6 mm, EPL 0.1%, LY30 0.1%, and CI 1.4). On postoperative day 1 (March 13, 2024), conventional coagulation tests were also within the reference ranges (PT 14.1 s, PTA 89.0%, INR 1.08, APTT 36.7 s, fibrinogen 3.31 g/L, and TT 15.2 s). Repeat thromboelastography on March 16, 2024 showed R 5.1 min, K 1.3 min, EPL 0.3%, and LY30 0.2% within the reference ranges, with increased angle (74.3°), MA (79.1 mm), and CI (3.8), indicating a postoperative hypercoagulable tendency rather than hypocoagulability. Therefore, an intraoperative coagulation abnormality cannot be directly assessed because no intraoperative coagulation test was obtained; however, the available preoperative and postoperative laboratory data did not demonstrate a bleeding-type coagulopathy. At the end of the operation, one drainage tube was placed in the splenic fossa and one in the pelvic cavity.

### 2.5 Pathologic examination

Gross examination of the splenectomy specimen showed one spleen measuring 22.2 cm × 15.8 cm × 6.2 cm. The capsule was smooth in some areas and rough in others. The cut surface was gray-red to gray-yellow. Some areas had a sponge-like appearance with visible hemorrhage and soft texture.

Microscopically, the splenic architecture was destroyed. The tumor was composed of abnormal vascular channels of variable size that anastomosed with one another. Some vascular channels were narrowed. Papillary projections were seen within the lumina. Focal areas showed solid growth. The tumor cells were round to oval. Some cells were enlarged, with irregular nuclear membranes and coarse chromatin. Extensive necrosis was present. Immunohistochemistry showed CD31 (diffuse positive), CD34 (positive), Ki-67 (labeling index approximately 40% positive), p53 (mutant-type expression pattern), INI-1 (diffuse positive), D2-40 (negative), EMA (negative), desmin (negative), SMA (negative), S-100 (focal positive), STAT6 (focal positive), FLI-1 (diffuse positive), ERG (positive), and HHV8 (negative).

The pathology report concluded that the findings supported a malignant vascular tumor. Considering the morphology of the resection specimen, positivity for vascular endothelial markers, a high proliferative index, mutant-type p53 expression, extensive necrosis, rupture with hemorrhage, and the clinical course, the pathologic diagnosis was splenic angiosarcoma (**Figure 2**).

**Figure 2:**
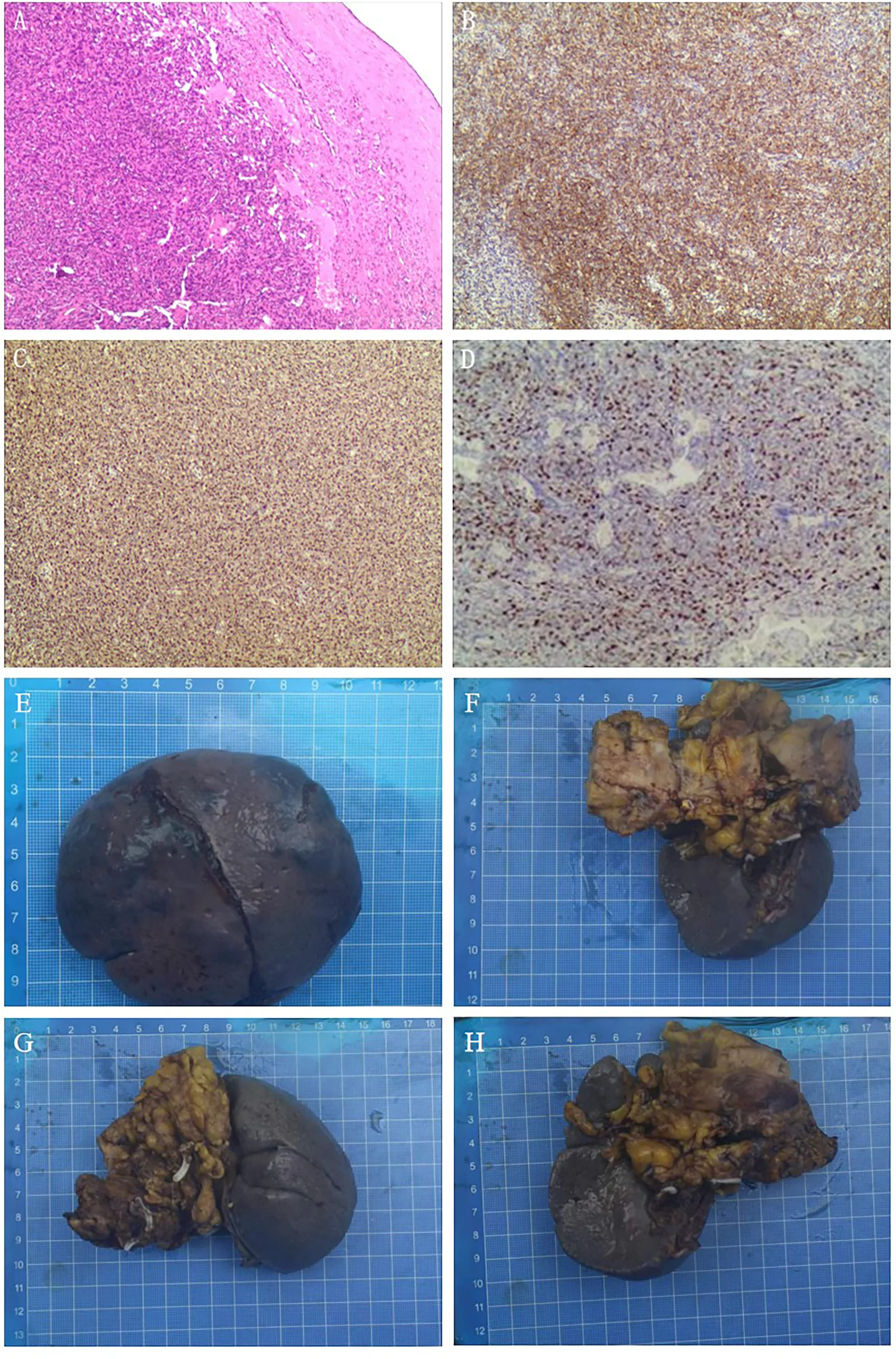
Pathological features of the splenic tumor. **(A)** H&E staining shows an infiltrative vascular tumor with irregular vascular channels and focal solid areas. **(B)** CD34 is positive in tumor cells. **(C)** FLI-1 shows diffuse nuclear positivity. **(D)** Ki-67 shows a high proliferative index of approximately 40%. **(E–H)** Gross photographs of the resected spleen from different views, showing the macroscopic appearance of the surgical specimen.

A supplementary pathology report on September 4, 2024 showed positive PD-L1 immunohistochemistry (SP263, Ventana BenchMark ULTRA), with a combined positive score of approximately 30. HER2 was 0. These supplementary findings were mainly relevant to treatment decisions. PD-L1 positivity provided a biological rationale for later treatment with a PD-1/PD-L1 inhibitor combined with anti-angiogenic therapy. HER2–0 did not support HER2-targeted treatment. The PD-L1 immunohistochemical analysis was performed as supplementary testing at an external pathology center rather than as part of the initial diagnostic panel. After splenectomy, our team recommended external supplementary pathologic testing, including PD-L1 immunohistochemistry; however, the patient initially preferred to defer additional testing at another institution. During subsequent systemic treatment, anlotinib had already been introduced as part of the second-line regimen. When immune checkpoint inhibitor therapy, particularly in combination with ongoing anti-angiogenic targeted therapy, was later being considered, the patient agreed to undergo the external supplementary pathological evaluation. Therefore, the approximately six-month interval between surgery and the September 4, 2024 supplementary report mainly reflected the timing of the patient’s decision to proceed with external testing rather than a prolonged laboratory turnaround time. After the report became available, immune checkpoint inhibitor therapy was initiated promptly: sintilimab was administered on September 13, 2024, nine days after the report.

### 2.6 Postoperative course, systemic treatment, and follow-up

Postoperative blood counts, biochemical tests, and inflammatory markers showed leukocytosis, anemia, hypoproteinemia, and electrolyte disturbance. On March 17, 2024, the amylase level in the drainage fluid was 11.5 U/L, with no evidence of pancreatic fistula. Enhanced CT on March 20, 2024 showed decreased left pleural effusion and a small right pleural effusion. After splenectomy, drainage tubes remained in place, and exudation, fluid collection, soft tissue swelling of the abdominal wall, and a small amount of gas were present in the operative area. Hepatic low-density lesions were more numerous than before, and some had enlarged. Some bowel loops contained fluid and gas with multiple air-fluid levels. After anti-infective therapy, inhibition of pancreatic enzyme secretion, nutritional support, and symptomatic treatment, the patient was discharged in stable condition on March 28, 2024. At discharge, his general condition was acceptable. The surgical incision had healed, and the sutures had been removed.

After surgery, the patient was transferred to the oncology department for further treatment. Based on chemotherapy summaries, imaging reports, and discharge records, the postoperative systemic treatment course is summarized in **Table 1**.

**Table 1:** Postoperative systemic treatment course.

Treatment phase	Time	Drugs and doses
Bone lesion-related treatment (denosumab)	Started on May 8, 2024; administered every 28 days or monthly.	Denosumab 120 mg, subcutaneous injection; used for prevention of skeletal-related events/bone protection in the setting of suspected bone metastases.
First-line chemotherapy: EIM regimen, cycle 1	May 9, 2024.	Ifosfamide (IFO) 2.0 g; epirubicin (EPI) 100 mg; mesna 0.4 g.
First-line chemotherapy: EIM regimen, cycle 2	June 4, 2024.	Ifosfamide (IFO) 2.0 g; epirubicin (EPI) 100 mg; mesna 0.4 g.
Second-line treatment: gemcitabine plus albumin-bound paclitaxel combined with anlotinib	June 26, 2024.	Gemcitabine 1,000 mg/m² (actual recorded doses: 1.8 g and 1.6 g on days 1 and 8); albumin-bound paclitaxel 125 mg/m² (actual recorded dose: 200 mg on days 1 and 8); anlotinib 8 mg orally once daily on days 1–14.
Second-line treatment: gemcitabine plus albumin-bound paclitaxel combined with anlotinib	July 20, 2024.	Gemcitabine 1,000 mg/m² (actual recorded doses: 1.8 g and 1.6 g on days 1 and 8); albumin-bound paclitaxel 125 mg/m² (actual recorded dose: 200 mg on days 1 and 8); anlotinib 8 mg orally once daily on days 1–14.
Second-line treatment: gemcitabine plus albumin-bound paclitaxel combined with anlotinib	August 11, 2024.	Gemcitabine 1,000 mg/m² (actual recorded doses: 1.8 g and 1.6 g on days 1 and 8); albumin-bound paclitaxel 125 mg/m² (actual recorded dose: 200 mg on days 1 and 8); anlotinib 8 mg orally once daily on days 1–14.
Second-line treatment: gemcitabine plus albumin-bound paclitaxel combined with anlotinib	Started on September 6, 2024; sintilimab was recorded as being administered on day 8, September 13, 2024.	Gemcitabine 1,000 mg/m² (actual recorded doses: 1.8 g and 1.6 g on days 1 and 8); albumin-bound paclitaxel 125 mg/m² (actual recorded dose: 200 mg on days 1 and 8); anlotinib 8 mg orally once daily on days 1–14; sintilimab 200 mg by intravenous infusion, actually administered on day 8.
Initiation of immunotherapy	September 13, 2024.	Sintilimab 200 mg by intravenous infusion; the chemotherapy record listed this as the actual day-8 administration during the gemcitabine plus albumin-bound paclitaxel combined with anlotinib regimen that started on September 6, 2024.
Transition to targeted therapy	September 28, 2024.	Anlotinib 8 mg.
Early stage of targeted therapy combined with immunotherapy	October 5, 2024.	Anlotinib 8 mg orally once daily on days 1–14; sintilimab 200 mg by intravenous infusion on day 1; denosumab 120 mg subcutaneously on the same day.
Maintenance targeted therapy combined with immunotherapy	October 25, November 17, and December 10, 2024; January 3, January 25, February 16, March 11, April 2, April 23, May 16, June 7, June 29, July 19, August 29, September 19, and October 10, 2025.	Anlotinib 8 mg orally once daily on days 1–14; sintilimab 200 mg by intravenous infusion on day 1; 21 days per cycle.
Continued treatment during the last documented hospitalization	Admitted on October 9, 2025, and discharged on October 10, 2025; the chemotherapy record listed the treatment start date as October 10, 2025.	Anlotinib 8 mg orally once daily on days 1–14; sintilimab 200 mg by intravenous infusion on day 1. The medical record documented an ECOG performance status score of 1, height of 170 cm, body weight of 65 kg, and body surface area of 1.71 m².

## 3 Discussion

### 3.1 Epidemiology and diagnostic challenges

PSA is extremely rare. World reviews, pathology series, SEER studies, and recent case reports show that common clinical clues include abdominal pain, splenomegaly, anemia, and splenic rupture. Patients may also present because of tumor metastasis, pancytopenia, or hemorrhagic shock (1–8). The present patient was 42 years old at onset. This finding suggests that even in younger patients, a vascular splenic lesion with a tendency toward bleeding or rupture should not be managed simply as a benign lesion.

Definitions of a ‘rare cancer’ vary across jurisdictions. As summarized by Sandler et al., the U.S. National Cancer Institute defines a rare cancer as one with an annual incidence of fewer than 15 cases per 100,000 persons, whereas the widely used European RARECARE definition applies a threshold of fewer than 6 cases per 100,000 persons per year (19, 20). These thresholds should therefore be specified when epidemiologic data from different regions are compared. Although each individual entity is uncommon, rare cancers collectively represent a substantial disease burden: the RARECARE analysis estimated approximately 541,000 new rare-cancer diagnoses annually in Europe (22% of all cancer diagnoses) and approximately 4.3 million prevalent cases (24% of total cancer prevalence) (20). PSA is far below either incidence threshold, with a reported annual incidence of approximately 0.14-0.23 cases per million population (5). This extreme rarity contributes to the limited population-level evidence and the absence of disease-specific prospective trials, so current knowledge remains largely dependent on pathology series, retrospective studies, and case reports.

The diagnostic process in this case is representative. Imaging initially interpreted the lesion as multiple splenic hemangioma-like lesions with local rupture and hemorrhage. Later, PET/CT suggested possible lymphoma because of high splenic metabolic activity, bone abnormalities, and increased pleural metabolic activity. Bone marrow examination did not support definite infiltration by a hematologic malignancy. Ultimately, the complete splenectomy specimen provided the diagnostic basis. This process shows that preoperative diagnosis of PSA often requires repeated correlation among imaging findings, clinical presentation, and pathology.

### 3.2 Imaging differential diagnosis

Both PSA and LCA can show complex imaging appearances. On CT, LCA may present as multiple low-density nodules in the setting of splenomegaly and may show progressive enhancement (21). On contrast-enhanced CT, PSA may present with splenomegaly, single or multiple nodules, mixed cystic-solid density, hemorrhage, necrosis, and heterogeneous enhancement. Patients may also have hepatic, bone, or pulmonary lesions. After summarizing the imaging findings of PSA, Thompson et al. suggested that PSA should be considered on imaging when a splenic mass is accompanied by evidence of metastasis (22). Therefore, when a splenic lesion is large, multifocal, hypervascular, and internally heterogeneous, benign hemangioma, LCA, lymphoma, metastasis, and PSA should all be included in the differential diagnosis.

In the present case, early contrast-enhanced CT described the hepatic lesions as hemangioma-like lesions. However, later records showed increases in the number and size of these lesions, and also documented shrinkage or partial shrinkage after systemic treatment. This clinical course supports the possibility of metastatic lesions. Therefore, this manuscript describes the hepatic findings as “hemangioma-like lesions; metastasis cannot be excluded; subsequently assessed as metastatic.” PSA may show high metabolic activity on FDG PET/CT and may be confused with lymphoma. Previous cases also indicate that the final diagnosis still requires pathologic support (23).

### 3.3 Pathologic diagnosis

The most important diagnostic basis in this case was pathology. The tumor destroyed the splenic parenchyma and formed abnormal vascular channels of variable size that anastomosed with one another. It also showed intraluminal papillary projections, focal solid growth, cellular atypia, increased mitotic activity, and extensive necrosis. These changes support splenic angiosarcoma (2, 24). Positivity for CD31, FLI-1, and ERG supports endothelial differentiation. ERG is one of the more sensitive vascular endothelial markers (25). HHV8 negativity does not support Kaposi sarcoma. D2–40 negativity does not support obvious lymphatic endothelial differentiation. Negativity for EMA, desmin, and SMA helps exclude epithelial, myogenic, and some spindle cell tumors.

The pathology report stated that the tumor supported a vascular origin. Because of cytologic atypia, increased mitotic activity, high proliferative activity, mutant-type p53 expression, extensive necrosis, and clinically aggressive behavior with rupture and hemorrhage, the pathologic diagnosis in this manuscript is stated as splenic angiosarcoma.

Microscopically, the tumor in this case consisted of anastomosing abnormal vascular channels, intraluminal papillary structures, and focal solid areas. These findings supported a malignant vascular tumor of the spleen. Immunohistochemistry showed diffuse CD31 positivity, diffuse FLI-1 positivity, and ERG positivity, supporting vascular endothelial differentiation. CD34 positivity was compatible with malignant vascular differentiation, and the high Ki-67 labeling index, mutant-type p53 expression, extensive necrosis, and rupture with hemorrhage supported malignant behavior.

Because some morphologic features overlapped with littoral cell-related tumors, littoral cell angioma and littoral cell angiosarcoma were retained in the differential diagnosis. The literature includes reports of malignant or metastasizing littoral cell-related tumors (12–18), supporting the need to distinguish these entities from PSA by integrating morphology, immunohistochemistry, and clinical behavior. The majority of reported littoral cell tumors are conventional littoral cell angiomas (LCAs), which are generally benign; in contrast, littoral cell hemangioendothelioma has been described as a rare low-grade or potentially malignant lesion, and littoral cell angiosarcoma (LCAS) is rare and overtly malignant (12–18, 24, 26, 27). Mou et al. describe LCA as an extremely uncommon benign splenic vascular tumor and illustrate that definitive diagnosis was established pathologically after splenectomy (26). A review of 180 reported littoral cell tumors by Sarandria et al. similarly identified LCA as the more common, typically benign entity, with littoral cell hemangioendothelioma and LCAS representing less common lesions with malignant potential (27). Because the clinical and imaging appearances of splenic vascular tumors overlap substantially, definitive classification requires histopathologic evaluation of tissue, with integration of architecture, cytologic atypia, proliferative activity, necrosis, and immunophenotype rather than reliance on imaging alone (17, 18, 24, 26, 27). Conventional LCA typically shows relatively bland sinus-like vascular channels and dual endothelial-histiocytic differentiation, whereas marked nuclear atypia, solid growth, high proliferative activity, extensive necrosis, and destructive behavior favor a malignant vascular neoplasm (9–18, 24, 27). Accordingly, distant metastases make conventional benign LCA substantially less likely and should heighten concern for angiosarcoma or another malignant littoral-cell tumor; however, metastasis alone does not establish a specific subtype because littoral cell hemangioendothelioma can also metastasize (13–15). In the present case, marked cytologic atypia, a Ki-67 labeling index of approximately 40%, mutant-type p53 expression, extensive necrosis, rupture with hemorrhage, and suspected hepatic and osseous involvement were inconsistent with conventional benign LCA. Moreover, the reported immunohistochemical panel documented endothelial differentiation (CD31, FLI-1, and ERG) but did not document the characteristic dual endothelial-histiocytic immunophenotype expected for conventional LCA. Taken together, the resection morphology, immunophenotype, and aggressive clinical behavior supported the final diagnosis of splenic angiosarcoma.

Supplementary pathology showed a PD-L1 combined positive score of approximately 30. This result should not be used to establish the diagnosis of PSA. Its relevance is mainly therapeutic. Previous studies suggest that PD-L1 may be expressed in some angiosarcomas (28). Therefore, the later use of a PD-1 inhibitor combined with anti-angiogenic therapy in this patient had a biological rationale, but PD-L1 positivity does not guarantee response.

### 3.4 Systemic treatment and treatment response in this case

There are no disease-specific prospective clinical trials for PSA. Systemic treatment for advanced cases is usually guided by experience with angiosarcoma in general and with soft tissue sarcoma. The ESMO guideline for soft tissue and visceral sarcomas provides an overall framework for systemic treatment of advanced soft tissue sarcoma, but it does not establish a separate standard regimen for PSA (29). Previous reports on systemic therapy for PSA also emphasize that evidence for splenic angiosarcoma is limited and that treatment often needs to be individualized (30).

Angiosarcoma may show some sensitivity to chemotherapy. Weekly paclitaxel has shown activity in unresectable or metastatic angiosarcoma (31). Anthracycline-based regimens and paclitaxel regimens have both been used for metastatic angiosarcoma (32). Retrospective evidence also supports the use of gemcitabine in advanced angiosarcoma (33, 34). In this case, the patient first received the EIM regimen containing ifosfamide and epirubicin. The EIM regimen included ifosfamide (IFO) 2.0 g, mesna 0.4 g, and epirubicin (EPI) 100 mg. After two cycles, the hemangioma-like hepatic lesions had enlarged. Metastasis could not be excluded, and the lesions were subsequently assessed as metastatic. The treatment response was assessed as PD.

The patient then received second-line treatment with gemcitabine plus albumin-bound paclitaxel combined with anlotinib; this combination was recorded as the GT regimen in the medical record. After two cycles of this regimen, the intrahepatic lesions, which were being assessed as metastatic, were broadly similar to previous images, and the response was assessed as SD. Follow-up on August 31, 2024 was also recorded as SD.

Anlotinib is a multi-target tyrosine kinase inhibitor. Its targets include VEGFR, PDGFR, FGFR, and c-Kit. It has an evidence base in bone and soft tissue sarcoma (35). The ALTER-S006 study evaluated anlotinib as maintenance treatment after first-line chemotherapy for advanced soft tissue sarcoma (36). A 2024 phase II study also evaluated first-line anlotinib in patients with soft tissue sarcoma who were not suitable for chemotherapy (37). A 2025 systematic review and meta-analysis suggested that anlotinib has a certain disease control rate in advanced bone and soft tissue sarcomas, although the evidence remains limited by study heterogeneity and the relatively high proportion of non-randomized studies (38). Therefore, the use of anlotinib in this case can be regarded as individualized PSA treatment based on experience with soft tissue sarcoma.

Evidence for immunotherapy in angiosarcoma is also increasing. A retrospective study by Ravi et al. suggested that some patients with angiosarcoma may benefit from immune checkpoint inhibitors (39). In the SWOG S1609 DART study, nivolumab plus ipilimumab showed activity in the cohort of patients with unresectable or metastatic angiosarcoma (40). Xu et al. reported a patient with metastatic PSA and high PD-L1 expression who achieved complete remission after anti-PD-1 therapy combined with anti-VEGF therapy (41). In the present case, the PD-L1 combined positive score was approximately 30, and the patient subsequently received anlotinib plus sintilimab. Imaging on October 25, 2024 showed partial shrinkage of hepatic lesions, indicating short-term imaging control with this regimen. Treatment response was assessed as SD at follow-up points in January, May, and August 2025. We hope that this treatment experience may provide a reference for future PSA management.

The patient also received denosumab. This manuscript describes the treatment background according to the imaging reports. CT on February 19, 2024 showed small patchy low-density areas with possible bone destruction in parts of the thoracolumbar vertebrae, some right ribs, and the right iliac bone. Whole-body bone scintigraphy on February 22, 2024 showed active bone metabolism in the right third anterior rib and left ribs, as well as multiple low-density bone lesions throughout the body, which were considered tumor-related. Later enhanced CT reports repeatedly described uneven bone density in parts of the ribs, spine, and pelvis, with metastasis considered or clinical follow-up recommended.

Based on these imaging findings of bone lesions, denosumab 120 mg was started by subcutaneous injection on May 8, 2024 and was subsequently administered approximately every 28 days or monthly. Denosumab was used to prevent skeletal-related events related to bone metastasis. The follow-up imaging description that “some thoracic vertebrae, parts of the bilateral ribs, and the thoracolumbar vertebrae showed uneven bone density, broadly similar to the previous scan on May 15, 2025” can be used to state that no definite imaging progression of bone lesions was recorded during denosumab treatment. Follow-up CT suggested overall stability of the bone lesions, and no definite new skeletal-related events were recorded. However, the single-agent effect of denosumab cannot be independently evaluated. Evidence for denosumab in solid tumor bone metastasis mainly concerns prevention or delay of skeletal-related events, rather than radiologic tumor shrinkage as a primary efficacy endpoint (42, 43). (**Table 2**) Importantly, the timeline shows that imaging evidence suspicious for osseous metastases was already present in February 2024, before splenectomy on March 12, 2024, whereas bone-directed treatment with denosumab was initiated postoperatively on May 8, 2024.

**Table 2:** Main post-treatment imaging follow-up and clinical efficacy assessment recorded in the medical record.

Time	Main imaging findings	Response assessment
June 2024, after two cycles of the EIM regimen	Re-examination after two cycles of first-line EIM chemotherapy showed enlargement of hemangioma-like hepatic lesions compared with previous imaging. Metastasis could not be excluded, and the lesions were subsequently assessed as possible metastatic disease.	PD
September 2, 2024	Multiple nodular low-density lesions were seen in the liver. The largest lesion measured approximately 2.8 cm. Some lesions showed ring enhancement, and some showed progressive enhancement. Compared with the previous imaging on August 9, 2024, the hepatic low-density lesions were largely similar in size, and some lesions showed necrotic changes. Metastasis was considered possible.	SD
October 25, 2024	Multiple nodular low-density lesions remained visible in the liver. The largest lesion measured approximately 2.8 cm. Compared with the previous imaging on September 2, 2024, the hepatic low-density lesions showed reduced density; some lesions had decreased in size, while the remaining lesions were largely unchanged.	SD
January 2, 2025	Multiple nodular low-density lesions remained visible in the liver. The largest lesion measured approximately 2.8 cm. Compared with the previous imaging on October 25, 2024, some hepatic nodules had decreased in size. Metastasis was still considered possible.	SD
May 15, 2025	The formal report showed multiple tiny nodules in both lungs, largely unchanged compared with January 2, 2025. Multiple nodular low-density lesions were present in the liver, and the largest lesion measured approximately 2.2 cm.	SD
August 8, 2025	The report showed persistent multiple nodular low-density lesions in the liver. The largest lesion measured approximately 2.2 cm.	SD

### 3.5 Prognosis and follow-up

The overall prognosis of PSA is poor. Previous pathology series, world reviews, SEER studies, and recent case reports indicate that the disease is often diagnosed at an advanced or metastatic stage. Splenic rupture, distant metastasis, large tumor size, and delayed diagnosis may be associated with poor outcomes (1–8). In this case, splenic rupture with hemorrhage was already present at onset. Preoperative imaging suggested bone lesions. After surgery, the patient was clinically managed as having stage IV disease with hepatic and bone involvement. These data suggest highly aggressive tumor biology in this case.

After multiple lines of systemic therapy, the patient achieved short-term imaging control. Follow-up on October 25, 2024 showed shrinkage of some hepatic lesions. The response after first-line EIM therapy was assessed as PD, whereas the response during second-line gemcitabine plus albumin-bound paclitaxel combined with anlotinib and later anlotinib plus sintilimab was recorded several times as SD. Formal imaging reports on January 2, 2025, May 15, 2025, and August 8, 2025 also included descriptions of partial lesion shrinkage. The last hospitalization record, dated October 10, 2025, showed that the patient was still receiving anlotinib plus sintilimab. His general condition at discharge was acceptable. Future follow-up should record skeletal-related events, pain, analgesic use, serum calcium, renal function, and dental or jaw-related adverse events to allow a more standardized evaluation of the benefit and safety of bone-protective treatment.

## 4 Conclusion

This case was classified as splenic angiosarcoma based on the resection specimen. Preoperative imaging suggested a vascular lesion at first and later possible lymphoma. Ultimately, pathology of the splenectomy specimen supported the diagnosis of PSA.

This case suggests that PSA should be considered early in complex vascular splenic lesions accompanied by splenomegaly, recurrent abdominal pain, anemia, and a tendency toward bleeding or rupture. In patients with an unclear diagnosis, complex lesions, or bleeding risk, splenectomy often has both therapeutic and diagnostic value. Advanced PSA still lacks a standard systemic treatment. In this case, short-term imaging control and partial shrinkage of hepatic lesions occurred during anlotinib plus sintilimab treatment. According to the medical record, the response at several later treatment points was assessed as SD. We hope that this treatment experience may provide a reference for future PSA management.

## Data Availability

The original contributions presented in the study are included in the article/supplementary material. Further inquiries can be directed to the corresponding authors.
